# Epilepsy as a dynamic disease: Toward actionable, individualized seizure risk prediction

**DOI:** 10.1111/epi.18602

**Published:** 2025-08-18

**Authors:** Kai Michael Schubert, Anthony G. Marson, Eugen Trinka, Marian Galovic

**Affiliations:** ^1^ Department of Neurology, Clinical Neuroscience Center University Hospital and University of Zurich Zurich Switzerland; ^2^ Institute of Systems, Molecular and Integrative Biology, Pharmacology, and Therapeutics University of Liverpool Liverpool UK; ^3^ Walton Centre NHS Foundation Trust Liverpool UK; ^4^ Department of Neurology, Neurocritical Care, and Neurorehabilitation Christian Doppler University Hospital, member of European Reference Network EpiCARE, and Center for Cognitive Neuroscience, Paracelsus Medical University Salzburg Austria; ^5^ Neuroscience Institute, Christian Doppler University Hospital Center for Cognitive Neuroscience Salzburg Austria; ^6^ Karl Landsteiner Institute for Clinical Neurosciences Salzburg Austria

**Keywords:** acquired epilepsy, COSY, individualized prediction, number needed to treat, prognostic models, seizure recurrence risk

## Abstract

The current definition of epilepsy allows diagnosis after a single unprovoked seizure if the estimated 10‐year recurrence risk is ≥60%. While this framework is grounded in epidemiological evidence, it does not align with the shorter time horizons that guide many clinical and personal decisions. In acquired epilepsies, such as those following stroke, traumatic brain injury, or CNS infections, most recurrences occur within 1–2 years, with risk declining sharply thereafter. This temporal clustering challenges the use of static, long‐term risk thresholds in isolation. Dynamic tools, such as the Chance of an Occurrence of a Seizure in the Next Year (COSY) and validated prognostic models (e.g., SeLECT, CAVE, RISE), offer recalculable, near‐term estimates that reflect evolving patient status. These metrics can improve communication, inform treatment thresholds through Number Needed to Treat (NNT) calculations, and enhance clinical trial recruitment by targeting periods of highest risk. However, barriers remain, including limited integration into guidelines, gaps in external validation, and the “Oedipus effect,” where probabilistic predictions influence patient behavior, treatment decisions, and research outcomes. Incorporating individualized, time‐sensitive risk prediction into clinical frameworks may better align diagnostic definitions with patient needs, reduce overtreatment, and optimize both everyday care and research in epilepsy prevention and management.

**Key points**

- Seizure risk after acquired brain insults peaks in the first 1–2 years; longer seizure‐free periods greatly reduce future recurrence odds.
- Near‐term seizure risk estimates (Chance of an Occurrence of a Seizure in the Next Year (COSY)) offer actionable insights beyond currently used 10‐year recurrence definitions.
- Prognostic models like SeLECT, CAVE, and RISE enable individualized seizure risk estimation after brain injury.
- COSY‐based number needed to treat calculations can set practical treatment thresholds and improve trial design.
- Risk predictions may alter patient behavior, treatment choices, and outcomes; the “Oedipus effect” highlights this complexity.

In 2005, the International League Against Epilepsy defined epilepsy conceptually as a “a disorder of the brain characterized by an enduring predisposition to generate epileptic seizures, and by the neurobiologic, cognitive, psychological, and social consequences of this condition.” This conceptual definition was operationalized in 2014, allowing a diagnosis after a single unprovoked seizure if the 10‐year recurrence risk is ≥60%—a threshold based on epidemiological data and comparable to risk after two seizures.^1^, ^2^ This captures elevated risk after events like stroke, traumatic brain injury, and central nervous system infections.

The 10‐year threshold of 60% recurrence risk is grounded in epidemiological data and may not address shorter time frames that drive day‐to‐day clinical decisions. In practice, patients and clinicians often focus on near‐term risk of recurrence—over days, months, or a few years—especially when navigating decisions related to social activities, driving, ability to work, private life decisions, treatment, or clinical trial participation.^3^, ^4^ This temporal disconnect may contribute to uncertainty and variability in clinical practice. To our knowledge, no formal studies have assessed the perspectives of patients and the public regarding the long‐term risk‐based approach. We argue here that integrating dynamic, short‐term risk estimates into existing frameworks could support more individualized decisions—particularly in the context of acquired causes.

## 1 THE CHALLENGE OF DIAGNOSING EPILEPSY BASED ON FUTURE RISK

The current definition allows diagnosing epilepsy after a single unprovoked seizure if the estimated 10‐year recurrence risk is ≥60%. Although this shift acknowledges advances in risk modeling and offers the potential for earlier intervention and development of disease‐modifying and antiepileptogenic therapies,^5^, ^6^, ^7^ it also introduces challenges; individuals may be labeled with epilepsy with all its psychosocial consequences despite never having another seizure.

A key limitation of this risk‐based approach is that seizure recurrence risk is dynamic and not constant over time. For example, an individual deemed to have a 60% recurrence risk immediately after a first poststroke seizure will have a substantially lower risk if they remain seizure‐free for several months. This evolving risk profile raises important questions; should a person who has been seizure‐free for 6 months after a single poststroke event still be considered to have epilepsy?

Using future risk to guide decisions about initiating antiseizure medications (ASMs) is conceptually sound. However, making a diagnosis of epilepsy that is based on long‐term risk does not mandate immediate antiseizure (ASM) treatment. Such decisions should also be informed by the degree to which ASM treatment is expected to reduce that risk, as well as the potential for adverse effects. Prior studies have shown that although early ASM treatment may reduce the likelihood of seizure recurrence, this benefit can be offset by medication adverse effects and the psychological and social burden of being labeled and treated for epilepsy.^8^

## 2 RETHINKING THE ROLE OF 10‐YEAR RISK

**FIGURE 1:**
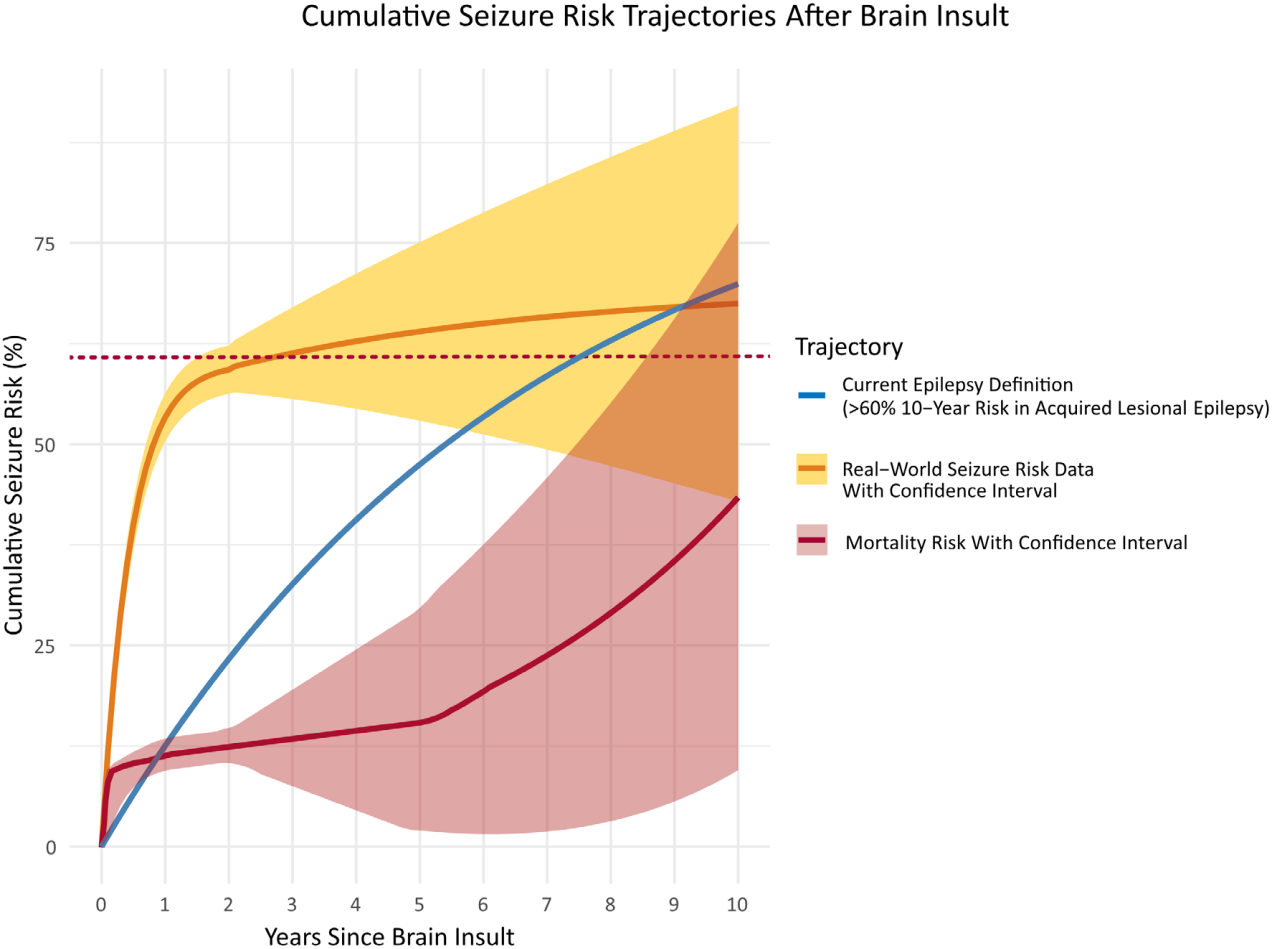
Conceptual cumulative risk trajectories for seizure recurrence and mortality following a brain insult, comparing the implied epilepsy definition with cohort‐based observations. This figure is a conceptual illustration based on the impression and interpretation of data from several published studies; it does not represent precise empirical estimates across all etiologies. The blue curve (“Current Epilepsy Definition”) reflects the diagnostic threshold allowing an epilepsy diagnosis after a single unprovoked seizure if the estimated 10‐year recurrence risk exceeds 60%. Although the definition specifies a cumulative probability over 10 years, it does not detail how risk accrues over time. For readers unfamiliar with seizure risk trajectories, this threshold could be misinterpreted as implying a steady, gradual increase in cumulative seizure probability across the decade. The orange curve (“Real‐World Seizure Risk”) is based on prognostic models and clinical cohort data. It shows that seizure risk is highest in the first 1–2 years postinsult—often reaching 60%–80%—and then slows considerably. This early clustering reflects the critical phase of epileptogenesis, but follow‐up data often become sparse over time, introducing increasing uncertainty (as reflected by widening confidence intervals). The red curve (“Mortality Risk”) captures the competing risk of death, which is rarely accounted for in seizure recurrence estimates. Mortality increases sharply in the early phase, slows down, and often rises again over longer time frames, particularly in older populations with acquired epilepsy (e.g., poststroke, traumatic brain injury, tumors). This trajectory becomes increasingly relevant with longer survival times, aging, and accumulating comorbidities.

In acquired epilepsy—particularly after stroke, trauma, or infections—the risk of a first unprovoked seizure is concentrated early after the initial insult. Multiple studies have shown that 60%–80% of such seizures occur within 2 years (Figure 1).^9^, ^10^, ^11^, ^12^, ^13^, ^14^, ^15^, ^16^, ^17^, ^18^, ^19^, ^20^, ^21^ Similarly, the risk of a second seizure is highest in the first 2 years after the initial seizure.^8^, ^12^, ^15^, ^19^, ^22^, ^23^, ^24^, ^25^ This clustering likely reflects either active epileptogenesis following the initial brain insult^26^ or an enduring predisposition^27^ established by the insult.^28^

Beyond this high‐risk period, seizure hazard typically declines. Patients who remain seizure‐free for 2 years after the index event have progressively lower odds of experiencing another seizure. The duration of seizure freedom is thus a strong predictor of short‐term risk. Additional factors—such as seizure cycles, medication changes, and comorbidities—also modulate risk over time. In older adults, this dynamic is further complicated by competing risks and higher mortality, which further erode the validity of long‐term projections.^29^, ^30^

This raises a key question: how can we better estimate seizure risk at the time when clinical decisions matter most, during the critical months and early years following a brain insult?

## 3 CHANCE OF OCCURRENCE OF A SEIZURE IN THE NEXT YEAR: A TOOL FOR MEANINGFUL SHORT‐TERM PREDICTION

**FIGURE 2:**
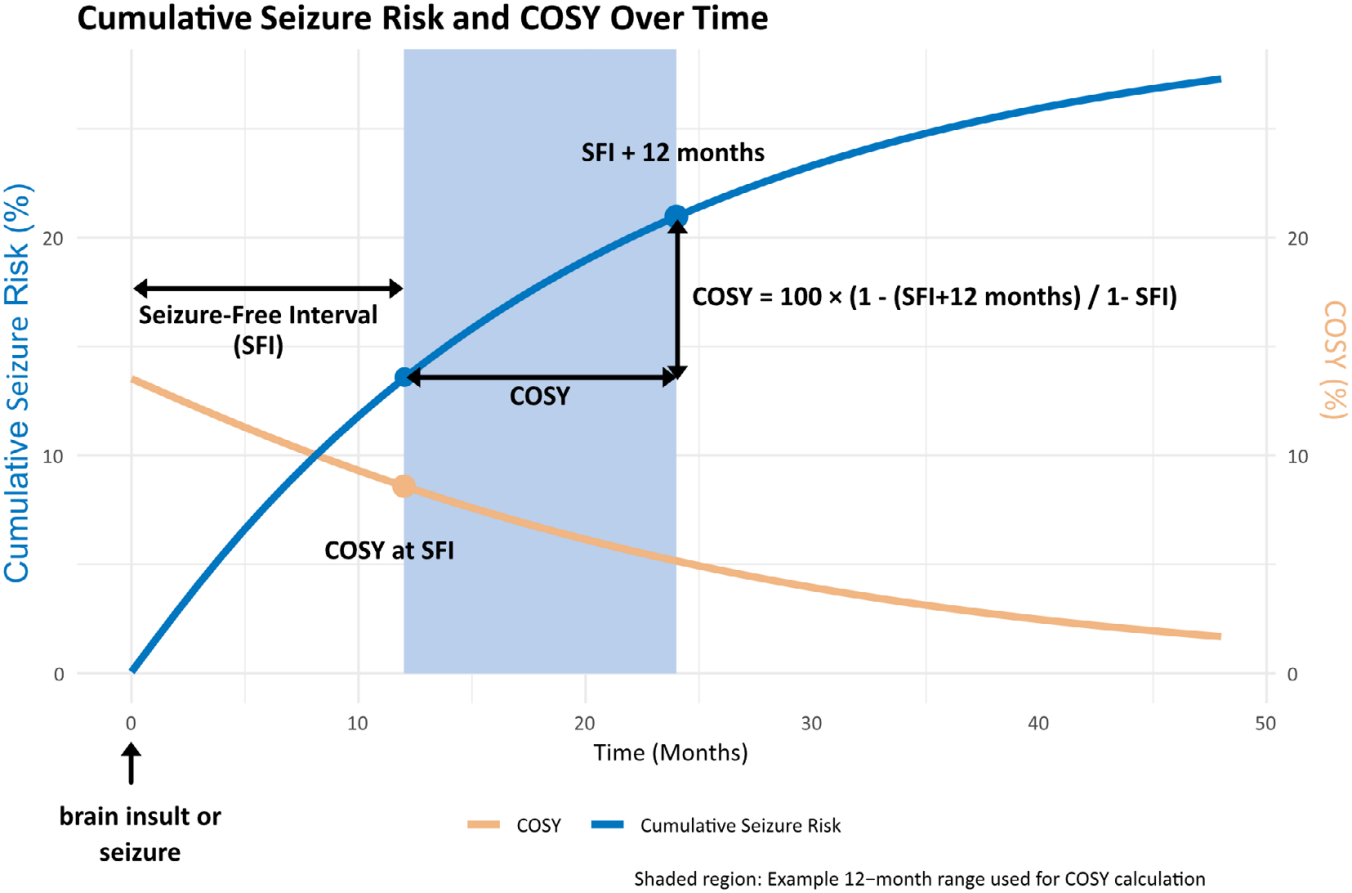
Chance of occurrence of a seizure in the next year (COSY). COSY is a metric developed to estimate the 1‐year probability of seizure recurrence based on prior seizure‐free duration.^32^ Originally introduced by the European Working Group on Epilepsy and Driving,^31^ COSY provides a standardized risk assessment tool that aids in clinical decision‐making and regulatory evaluations, such as driving eligibility.^23^, ^25^, ^33^, ^34^ COSY is calculated using cumulative seizure‐free probabilities from epidemiological studies and follows the formula COSY = 100 × (1 − SFI + 12 months / 1 − SFI), where SFI (seizure‐free interval) + 12 months represents the probability 12 months later. For example, if a person has an 86% probability of being seizure‐free at 12 months and a 78% probability at 24 months, their COSY value at 12 months is COSY = 100 × (1 − .78 / 1 − .86) ≈ 9%. This means there is a 9% chance of experiencing a seizure within the next year. COSY has been used to refine fitness‐to‐drive regulations and offers potential applications in clinical epilepsy risk assessment and treatment decisions.

The chance of occurrence of a seizure in the next year (COSY), developed by the European Working Group on Epilepsy and Driving,^31^ estimates the 1‐year risk of seizure recurrence based on time since last seizure.^32^ COSY (or its monthly counterpart, chance of occurrence of a seizure in the next month) offers a dynamic and recalculable metric measuring near‐term risk (Figure 2).

Unlike static 10‐year estimates, COSY adapts as time passes, enabling more individualized and time‐sensitive decisions, particularly after a first seizure. It also facilitates communication; whereas a 60% 10‐year risk is abstract, a 20% 1‐year risk is tangible and more useful for decisions on ASM initiation or discontinuation, driving eligibility, or return‐to‐work assessments.^23^, ^25^, ^33^, ^34^, ^35^

As seizure‐free intervals lengthen, COSY values decline. A high initial COSY may drop substantially after 6 months without recurrence, potentially calling into question whether the diagnosis of epilepsy still applies, and consequently whether treatment is still needed (Figure 2).

## 4 WHAT PROGNOSTIC MODELS CAN OFFER

To address the need for actionable, individualized, and evidence‐based seizure risk assessment, a number of prognostic models have emerged in recent years. Tools such as the SeLECT,^9^, ^36^, ^37^, ^38^, ^39^ CAVE,^16^ CAVE2,^40^ LANE,^41^ RISE,^17^ and DIAS^18^ models integrate clinical, imaging, and sometimes electrophysiological features to estimate seizure risk after brain injury (Table 1). Developed and validated across large multicenter datasets, they increasingly reflect real‐world patient diversity.^42^

**TABLE 1:** Prognostic models for predicting remote symptomatic seizures and epilepsy after acquired brain injury: patient groups, validation, variables, and applicability.

Model name	Patient group	Validation	Key variables	Apparatus tests	Calculation complexity	Calculator available	High‐risk identification (10‐year seizure risk > 60%)
SeLECT score^9^, ^34^, ^36^, ^37^, ^39^, ^60^	2023: derivation: 4552 patients, 9 international centers (2002–2019)	Internal: derivation cohort (9 international subcohorts) 2023 replication cohort for SE: 39 patients with poststroke SE, 3 separate cohorts	NIHSS score, large‐artery atherosclerosis, acute symptomatic seizures, cortical involvement, territory of MCA involvement	Yes (CT or MRI required)	Moderate (scores)	Yes	Yes
CAVE score^16^	Derivation: 993 patients (*n* = 764 >7‐day survivors), Helsinki (2005–2010) Validation: 325 patients, Lille (2004–2009)	Internal: retrospective cohort (University Hospital Helsinki) External: 325 patients, Lille (2004–2009)	Cortical involvement, age, volume, seizures	Yes (CT or MRI required)	Low to moderate (score with 4 variables)	No	No (risk of 4 points after 2.7 years only 47%)
CAVE2 score^40^	Derivation: 408 patients, Taiwan (2013–2019)	Internal: retrospective cohort (Ditmanson Medical Foundation Chia‐Yi Christian Hospital)	Cortex involvement, age, volume, seizures	Yes (CT or MRI required)	Low to moderate (score with 4 variables)	No	Yes
LANE score^41^	Derivation: 602 patients, Hospital of Qingdao University (2014–2017) Validation: 521 patients, Qingdao Municipal Hospital (2015–2017)	Internal: retrospective cohort (Affiliated Hospital of Qingdao University) External: 521 patients, Qingdao Municipal Hospital	Lobar hemorrhage, age, NIHSS score, seizures	Yes (CT or MRI required)	Low to moderate (score with 4 variables)	No	Yes
RISE score^17^	Derivation: 419 patients, Vall d'Hebron University Hospital (2012–2021) Validation: 308 patients, Bellvitge University Hospital (2011–2022)	Internal: retrospective cohort (Vall d'Hebron University Hospital) External: 308 patients, Bellvitge University Hospital	Premorbid mRS, VASOGRADE, surgical treatment, seizures	Yes (MRI required)	Low to moderate (score with 4 variables)	No	Yes
Brain metastases epilepsy risk score^61^	Derivation: 799 patients, Zurich (2004–2014)	Internal: retrospective cohort (University Hospital Zurich)	Supratentorial BM, incomplete resection, multiple surgeries	Yes (MRI required)	Low (score with 3 variables)	No	No (risk of 8 points after 5 years only 47.4%)
STAMPE2 score^62^	Derivation: 779 patients, Zurich (2000–2013)	Internal: retrospective cohort (University Hospital Zurich)	Sensorimotor deficit, tumor progression, age < 55 years, major surgical complication, preoperative epilepsy, epileptiform potentials on postoperative EEG, edema	Yes (MRI and EEG required)	Moderate (score)	No	No (no percentages, only expert recommendation)
Postoperative glioma‐related epilepsy model^63^	Derivation: 166 patients Validation: 42 patients, Beijing Tiantan Hospital	Internal: retrospective cohort (Beijing Tiantan Hospital)	Age, temporal lobe involvement, preoperative epilepsy, predictive genes	Yes (CT or MRI required)	Moderate to high (nomogram)	No	Yes
Posttraumatic epilepsy nomogram (1)^64^	Derivation: 1301 patients, West China Hospital (2011–2017) Validation: 834 patients, two cohorts (2013–2015)	Internal: retrospective cohort (West China Hospital) External: two independent cohorts (421 patients from Chengdu Shang Jin Nan Fu Hospital and 413 patients from Sichuan Provincial People's Hospital)	Loss of consciousness, subdural hematoma, contusion side, Glascow Coma Scale, early posttraumatic seizures, sex, treatment (conservative, puncture, surgery)	Yes (CT or MRI required)	Moderate to high (nomogram)	No	Yes
Posttraumatic epilepsy nomogram (2)^65^	Derivation: 457 patients, Qinghai Provincial People's Hospital (2016–2019)	Internal: retrospective cohort (Qinghai Provincial People's Hospital)	Contusion site, skull fracture, Glasgow Coma Scale, chronic alcohol use, contusion volume, subdural hematoma, nonlate posttraumatic seizures	Yes (CT or MRI required)	Moderate to high (nomogram)	No	Yes
DIAS3 score^18^	Derivation cohort: 1128 patients (International Cerebral Venous Thrombosis Consortium) Validation cohorts: 543 (ACTION‐CVT) and 556 (Israel CVT study); multinational, hospital‐based.	Internal validation with bootstrapping, external validation using two independent multicenter cohorts	Decompressive hemicraniectomy, intracerebral hemorrhage at baseline, age (in decades), seizures in acute phase (excluding SE), SE in acute phase, subdural hematoma at baseline	Yes (CT or MRI required)	Moderate to high (calculation table)	No	Yes

Abbreviations: BM, brain metastases; CT, computed tomography; CVT, cerebral venous thrombosis; EEG, electroencephalography; MCA, middle cerebral artery; MRI, magnetic resonance imaging; mRS, modified Rankin Scale; NIHSS, National Institutes of Health Stroke Scale; SE, status epilepticus; VASOGRADE, World Federation of Neurosurgical Societies (WFNS) grading scale for aneurysmal subarachnoid hemorrhage based on clinical status and Fisher grade.

Their key strength lies in predicting short‐ to medium‐term risk (1–5 years), aligning with seizure patterns after acquired injuries and better supporting clinical decision‐making, particularly in older adults, where long‐term projections are often unreliable due to competing mortality risks.

Despite their promise, these models remain underutilized in practice. They are not yet integrated into major guidelines, and their application may require some technical familiarity. To improve access, we developed https://predictepilepsy.com/, a platform that showcases several established risk models and includes user‐friendly COSY calculations.

## 5 REDEFINING CLINICAL THRESHOLDS: PROGNOSTIC MODELS, COSY, AND THE NUMBER NEEDED TO TREAT FRAMEWORK

A powerful application of prognostic models and COSY is in defining treatment thresholds through the lens of number needed to treat (NNT). In cardiovascular medicine, tools like the CHA_2_DS_2_‐VASc score guide anticoagulation based on stroke risk and expected treatment benefit, weighted against the risk of bleeding.^43^ Using COSY‐based calculations may enable a similar paradigm in epilepsy.

We illustrate the utility of the COSY‐based framework using candidate pharmacological interventions aimed at preventing epilepsy following a brain insult. Recent observational studies suggest that certain agents—such as specific antihypertensives,^44^, ^45^ statins,^46^ or glucagonlike peptide‐1 receptor agonists^47^—may reduce the risk of epilepsy by 23%–34% (Table 2). Applying relative risk reductions (RRRs) from these studies to individual COSY estimates allows calculation of clinically meaningful NNTs. Specifically, NNT can be approximated by the following formula:

$$\mathrm{NNT}\approx 1/\left(\text{COSY}\times \mathrm{RRR}\right)$$

where COSY represents the baseline 1‐year probability of seizure (expressed as a proportion) and RRR is the relative risk reduction achieved by the intervention.

**TABLE 2:** Potential repurposed medications for preventing remote symptomatic seizures/epilepsy after brain injury: reported relative risk reductions and cosy‐based number needed to treat.

Author	Title	Date of publication	Journal	Population analysis	Control	Statistical approach	RRR	Population size	Baseline COSY for NNT = 25	Baseline COSY for NNT = 50
Sindhu et al.	Newer Glucose‐Lowering Drugs Reduce the Risk of Late‐Onset Seizure and Epilepsy	2024	*Epilepsia Open*	Patients with diabetes, focus on GLP‐1 receptor agonists	Placebo	Meta‐analysis of RCTs	.24	~200 000	**16.67**	**8.33**
Wen et al.	Angiotensin Receptor Blockers for Hypertension and Risk of Epilepsy	2024	*JAMA Neurology*	Hypertensive patients, comparison of ARBs vs. ACEI, β‐blockers, and CCBs	ACEI, β‐blockers, CCBs	Retrospective cohort analysis with propensity score matching	.3	2 261 964	**13.33**	**6.67**
Hufthy et al.	Statins as Antiepileptogenic Drugs: Analyzing the Evidence and Identifying the Most Promising Statin	2022	*Epilepsia*	Poststroke or vascular disease populations, retrospective and preclinical analysis	No statins or other treatments	Retrospective clinical and preclinical studies	.34	Varies (retrospective and experimental models)	**11.76**	**5.88**
Doege et al.	Association Between Angiotensin Receptor Blocker Therapy and Incidence of Epilepsy in Patients With Hypertension	2022	*JAMA Neurology*	Hypertensive patients, general comparison of ARBs	ACEI, β‐blockers, CCBs	Retrospective cohort analysis	.23	168 612	**17.39**	**8.7**

Abbreviations: ACEI, angiotensin‐converting enzyme inhibitor; ARB, angiotensin receptor blocker; CCB, calcium channel blocker; COSY, chance of occurrence of a seizure in the next year; GLP‐1, glucagonlike peptide‐1; NNT, number needed to treat; RCT, randomized clinical trial; RRR, relative risk reduction.

Boldface indicates the baseline COSY (Chance of an Occurrence of a Seizure in the Next Year) values used for calculating the Number Needed to Treat (NNT) at specified thresholds.

**FIGURE 3:**
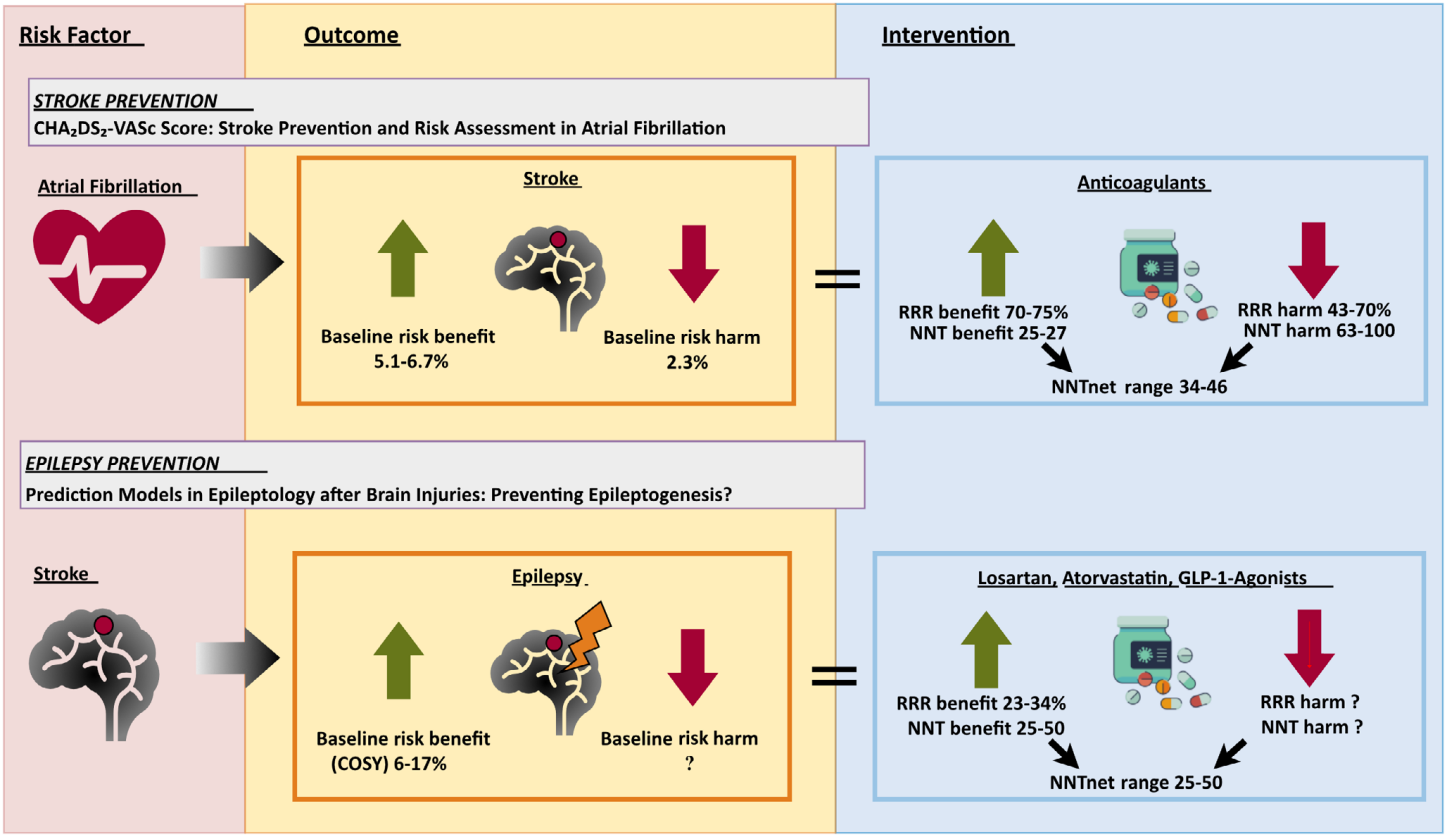
Number needed to treat (NNT) and net NNT (NNT_net) in stroke and epilepsy prevention. Figure 2 illustrates the concept of NNT in two clinical scenarios: stroke prevention in atrial fibrillation (AF)^43^ and epilepsy prevention (antiepileptogenesis) after stroke. In the upper part of the figure, anticoagulation is shown as a preventive strategy against stroke in AF patients. Several prospective trials, such as AMADEUS,^66^ RE‐LY,^67^ and ARISTOTLE,^68^ have established the efficacy and risks of anticoagulation in stroke prevention. Although it reduces the risk of ischemic stroke (relative risk reduction [RRR] benefit) by 65%–75%, it also increases the risk of major bleeding (relative risk increase) with an RRR harm of 43%–70%. To correctly assess the net benefit of anticoagulation, we must consider that the baseline risks for stroke and major bleeding are different. Because the risk reduction from anticoagulation applies to stroke prevention and the risk increase applies to bleeding, we use absolute risk reductions (ARRs) rather than RRRs to calculate NNT_net: NNT_net = 1/(ARR_benefit − ARR_harm), where ARR_benefit represents the absolute reduction in stroke risk with anticoagulation, and ARR_harm represents the absolute increase in bleeding risk. Based on real‐world data, NNT_net for anticoagulation in AF typically ranges between 34 and 46, meaning that for every 34–46 patients treated, one additional patient benefits when considering both stroke prevention and bleeding risks. This highlights the trade‐off; although anticoagulation is crucial for stroke prevention, it carries a bleeding risk and should be tailored to the individual's baseline stroke risk (e.g., estimated using the Calculator of Absolute Stroke Risk). In contrast, for antiepileptogenesis after stroke, retrospective studies suggest that antihypertensives, glucagonlike peptide‐1 (GLP‐1) receptor agonists, and statins may reduce poststroke epilepsy risk, with reported RRRs (RRR_benefit) of 23%–34%. Unlike anticoagulation, the associated harm (RRR_harm) is likely minimal or unknown, making the simpler formula applicable: NNT = 1 / (RRR × Risk_baseline), where Risk_baseline represents the estimated 1‐year probability of seizure without intervention. Because there is no known substantial harm from these interventions, NNT_net is assumed to be approximately equal to NNT_benefit, with estimates ranging from 25 to 50. A key limitation of current antiepileptogenesis research is that it is mostly retrospective, often reporting stronger effects than prospective trials. However, when tested in randomized settings, such effects often tend to be smaller. Thus, further prospective validation studies are essential to ensure chance of occurrence of a seizure in the next year‐based stratification are clinically meaningful.

For example, a baseline COSY of 10% with a 30% RRR yields an approximate NNT of 33; at a COSY of 20%, the NNT improves to approximately 17. If these effect sizes are confirmed in prospective randomized trials, the resulting NNTs would compare favorably with established preventive interventions in other areas of medicine (see Figure 3 for a detailed explanation and example calculation).

## 6 ENRICHING TRIALS, ADVANCING EPILEPTOGENESIS RESEARCH

COSY also provides a practical framework for improving trial design. Many antiepileptogenesis studies suffer from heterogeneous inclusion criteria and underpowered effect sizes.^7^, ^48^ COSY‐based thresholds enable focused recruitment based on estimated seizure risk.^49^

This is already underway. Recent trials,^50^ including one evaluating eslicarbazepine in poststroke patients, have employed SeLECT and CAVE models to enrich for high‐risk participants.^48^ COSY could refine this further, identifying a risk window where treatment is both most needed and most likely to demonstrate efficacy.

Coupled with existing models, COSY enables trial inclusion criteria to reflect both biological plausibility and practical utility, improving generalizability while maintaining rigor.

## 7 LIMITATIONS OF PROGNOSTIC MODELS

Despite their promise, prognostic models and COSY have limitations. Most are based on retrospective data with missing prospective validation, potentially leading to inaccurate risk estimates.^42^, ^48^

**FIGURE 4:**
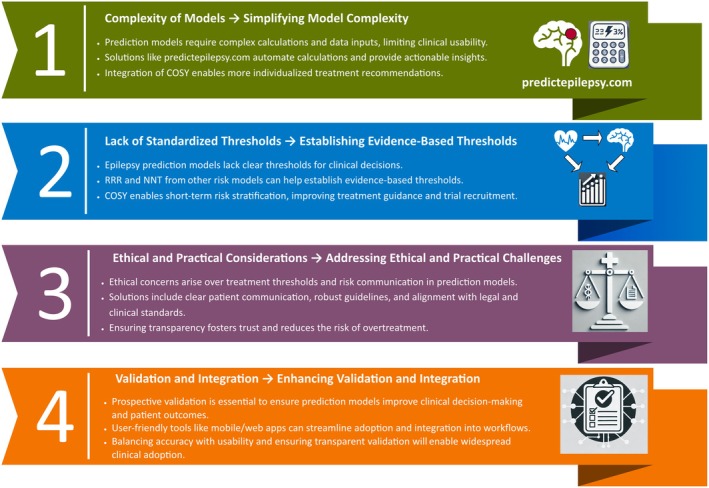
Key barriers and solutions for implementing seizure prediction models in clinical practice. Challenges include model complexity, lack of treatment thresholds, ethical concerns, and limited validation—all of which can be addressed through tools like predictepilepsy.com, chance of occurrence of a seizure in the next year (COSY)‐based risk framing, transparent communication, and prospective integration. NNT, number needed to treat; RRR, relative risk reduction.

Communication of probabilistic risk remains ethically and practically challenging, especially when treatment carries harms. Without clear guidance, clinicians may misinterpret or underuse model outputs. Transparency and adherence to standards like TRIPOD^51^, ^52^ or PROBAST^53^, ^54^ are needed to ensure quality and comparability yet remain inconsistently applied. Small, single‐center datasets and limited external validations also hinder generalizability. Future efforts must prioritize multicenter data, diverse populations, and robust integration into digital health systems to realize the full clinical utility of seizure prediction tools (Figure 4).^42^

Current models and definitions often omit seizure type^55^ (e.g., focal vs. generalized, with vs. without impaired consciousness) and timing (seizure from awake vs. from sleep)—factors that influence risk of disability, injury, mortality, and fitness for tasks like driving or operating machinery.

Finally, predictions can influence the outcomes they aim to forecast—a phenomenon known as the “Oedipus effect,” first described by philosopher Karl Popper in 1957.^56^ Named after the Greek myth^57^ in which a prophecy shapes events it foretells, this metaphorical reference in the context of epilepsy highlights how risk predictions and diagnostic labels—particularly when based on probabilistic models—can shape patient behavior, treatment choices, identity, and social outcomes.^58^ Although predictions do not cause epilepsy itself, they may indirectly influence observed trajectories through expectancy or labeling effects. Such a diagnosis can shape patient behavior, potentially encouraging health‐conscious decisions but also leading to avoidance behaviors, stigma, or social and occupational limitations.^59^ Second, physicians may adjust treatment strategies based on perceived relapse risk, potentially favoring early interventions after brain injury while reducing treatment later. Third, in research, predicted risks influence definitions of active versus resolved epilepsy. Even with accurate models, we must acknowledge that predictions can influence clinical decisions, patient behavior, and consequently, observed outcomes—making the overall impact of prediction inherently complex, even if the underlying disease course remains unchanged.

## 8 CONCLUSIONS: FROM RISK TO ACTION

Seizure risk prediction is central to epilepsy care and research. The current reliance on 10‐year recurrence estimates provides a robust diagnostic foundation, but it does not always match the clinical decisions that patients and providers face. Prognostic models and COSY represent complementary tools; they may improve clinical decision‐making by adding practical, near‐term risk assessments to existing definitions. These tools provide a dynamic, intuitive, and evidence‐based metric that supports treatment decisions, patient communication, and trial design. Although the data and examples presented here focus primarily on epilepsy following acquired brain insults, the framework of dynamic, individualized risk prediction may also prove valuable across other etiologies of epilepsy.

## AUTHOR CONTRIBUTIONS

Kai Michael Schubert and Marian Galovic conceived the idea and drafted the manuscript. Anthony G. Marson and Eugen Trinka critically reviewed and revised the manuscript. All authors approved the final version of the manuscript.

## CONFLICT OF INTEREST STATEMENT

M.G. has received fees and travel support from Arvelle, Advisis, Bial, and Nestlé Health Science outside the submitted work. E.T. has received personal fees from EVER Pharma, Marinus, Arvelle, Angelini, Argenx, Medtronic, Biocodex, Bial‐Portela & Cª, Newbridge, GL Pharma, GlaxoSmithKline, Boehringer Ingelheim, LivaNova, Eisai, Epilog, UCB, Biogen, Sanofi, Jazz Pharmaceuticals, and Actavis, and his institution has received grants from Biogen, UCB Pharma, Eisai, Red Bull, Merck, Bayer, the European Union, FWF Österreichischer Fond zur Wissenschaftsforderung, Bundesministerium für Wissenschaft und Forschung, and Jubiläumsfond der Österreichischen Nationalbank (none of them related to the presented work). The other authors declare no competing interests. We confirm that we have read the Journal's position on issues involved in ethical publication and affirm that this report is consistent with those guidelines.
